# Psychological Impact of COVID-19 Emergency on Health Professionals: Burnout Incidence at the Most Critical Period in Spain

**DOI:** 10.3390/jcm9093029

**Published:** 2020-09-20

**Authors:** José Ángel Martínez-López, Cristina Lázaro-Pérez, José Gómez-Galán, María del Mar Fernández-Martínez

**Affiliations:** 1 Department of Social Work and Social Services, University of Murcia, Avda. Teniente Flomesta, 5-30003 Murcia, Spain; jaml@um.es; 2 Department of Sociology, University of Murcia, C/Campus Universitario, 11, 30100 Murcia, Spain; cristina.lazaro2@um.es; 3 Department of Education, University of Extremadura, Avda. de Elvas, s/n, 06006 Badajoz, Spain; 4 College of Education, Ana G. Méndez University, Cupey Campus, San Juan, PR 00926, USA; 5 College of Education Sciences & College of Sociology, Social Work and Public Health, University of Huelva, Campus El Carmen, Avda. de las Fuerzas Armadas, s/n, 21007 Huelva, Spain; mar.fernandez@dstso.uhu.es

**Keywords:** burnout, COVID-19, pandemic, health professionals, stress, anxiety, prevention

## Abstract

Background: The health profession is a burnout producer due to the continuous contact with pain and suffering. In addition, excessive workloads can generate stress and psychological distress. Objectives: The aim of this study was to determine the degree of burnout and its main triggers in health professionals in Spain at the most critical period of the COVID-19 emergency. Method: A quantitative research was developed through a simple random sampling in different Spanish hospitals through the period of greatest impact of the pandemic (*N* = 157). Data were collected using a standardized questionnaire from Maslach burnout inventory (MBI) containing 22 items, which measures three subscales: emotional burnout, depersonalization, and self-fulfillment. Results: depersonalization values reached 38.9%. A total of 90.4% of the health professionals considered that psychological care should be provided from the work centers. Furthermore, 43.3% of the health professionals estimated that they might need psychological treatment in the future. Finally, 85.4% stated that the lack of personal protective equipment (PPE) generated an increase in stress and anxiety. Conclusion: This study demonstrates the need to consider specific mental health care services and training in crises to avoid possible psychological disorders. The information obtained is also valuable for the development of future prevention protocols and training of health personnel to face pandemics of these characteristics or emergency scenarios. Having the necessary physical means for their protection, as well to updated regular and accurate information, is essential to avoid feelings of fear and uncertainty. This would promote the health of these professionals.

## 1. Introduction

The expansion in March 2020 of the epidemic of the SARS-CoV-2 coronavirus, COVID-19, has meant an unknown scenario for the world’s population where panic and fear of contagion has spread rapidly to all continents as a reaction to globalization, taking over almost completely the information transmitted by the media and social networks [1,2,3,4]. The disease that began in Wuhan (China) in December 2019, initially associated with pneumonia, quickly became a global epidemic [5]. It became a major global problem, in such a way that on 30 January 2020 the World Health Organization designated the COVID-2019 outbreak as a “public health emergency of international concern” (PHEIC) because of its rapid spread [6].

This pandemic has changed the lives and behaviors of all people (habits, customs, ways of relating, confinement in the digital age, etc.) [7,8] and professionals (health protocols, health alerts, isolation measures, individual and community prevention measures) [9,10]. A single cause has provoked the same collective thinking at a global level and is based on two main lines of action; first, the need to protect ourselves from the virus, and second, to resist the social and economic crisis caused by it.

The actions aimed at surviving the COVID-19 disease also involved managing personal skills and resources to combat the secondary effects that this pandemic may have produced. As many authors have already pointed out, the pandemic has had psychological effects on the general population, but especially on health professionals [11,12,13,14]. This group has been exposed for a long period of time to the constant threat of infection with the virus, which is often described as fatal, and which causes a sense of danger and uncertainty in their daily activities among health workers and also in society as a whole [15].

According to the Report on the situation of COVID-19 in health care personnel in Spain, as of 21 May 2020, 40,921 had been infected and 53 deaths had been reported through the SiViES platform of the Centro Nacional de Epidemiología (National Center for Epidemiology) [16]. Considering that the total number of people infected in Spain on that same date was 269,863 [17], the number of health professionals infected exceeds 15%, a sign of the risk to which they have been exposed in their professional work. However, these data should be considered with caution since the number of figures has constantly changed. Updated data from May 29 of the same report shows 40,961 positive cases in health professionals and 52 deaths, fewer than previous counts.

This official report does not include the characteristics related to the professional category of health professionals or other considerations related to professional practice. On 9 May 2020, the Asociación de Médicos Unidos por sus Derechos (Physicians United for their Rights of Association) presented the report “Condiciones de los médicos españoles en la práctica clínica durante la crisis de COVID-19” (conditions of Spanish physicians in clinical practice during the COVID-19 crisis), which included higher figures than those provided by the Ministry, with 48,320 health professionals infected and 76 dead [18]. In addition, this report includes elements that show how health professionals have dealt with the pandemic and under what conditions.

The most relevant data shows that 12% of the physicians had suffered from the disease (data without taking into account those who were asymptomatic); 23% of the physicians suffering from COVID-19 had returned to work without having performed PCR (polymerase chain reaction), that is, without checking through the corresponding diagnostic tests if they were free of the virus or if they had developed antibodies, which would help prevent the virus from spreading in hospitals. Those measures would have help them to prevent the virus from spreading in hospitals; 66.5% had attended patients with COVID-19, 49% considered that they lacked sufficient protective material, highlighting the fact that 1/3 did not have Fpp2 or Fpp3 masks, and one in four physicians had reused their mask for more than a week. Finally, 86.8% of physicians considered that they could have been a vector of the disease because they lacked adequate protective material [18].

This amount of numbers causes uncertainty in the population, especially among health professionals. Altogether, with the lack of personal protective equipment (PPE) and the contradictory information, due to the lack of knowledge of the virus by official sources, are elements that keep the level of professional anxiety very high, and may cause other disorders that would not benefit professional practice at all.

In this scenario, health professionals have carried out a titanic task, with personal unprecedent, work, and social pressure within a context of insufficient PPE to fight this disease. Recent studies [19,20,21,22] show how working under pressure from COVID-19 have a significant impact on health professionals, especially those who work in a more precarious context, working long hours, without adequate PPE, etc. In addition, Lai et al. point out that in Wuhan “a considerable proportion of health workers reported experiencing symptoms of depression, anxiety, insomnia and distress, especially women, nurses and frontline health care workers directly involved in the diagnosis, treatment or nursing care of patients with suspected or confirmed COVID-19” [23]. Due to the fact that during the stages prior to the health crisis, in previous studies carried out in Spain, medium-high values of burnout were found [24,25,26,27,28] we asked whether during this phase of increased active cases and overflow of health care facilities caused by the SARS-CoV-2 pandemic, professionals might exhibit evidence of the three components of the Maslach burnout scale [29], emotional exhaustion, depersonalization, and changes in self-fulfilment, as these are the ones that other studies have indicated are most at risk of developing health problems from COVID-19 [23,30]. At the same time, the perception of stress among professionals is closely related to burnout, reporting a higher level of emotional exhaustion and depersonalization [31]. While it is true that burnout is a process that develops over time [32], during the months of March and April the volume, workload and deaths of patients admitted were much higher than in previous months [33], which makes us wonder if this context could have aggravated the situation as occurred in other countries [34,35] in the three subscales of the Maslach burnout inventory.

## 2. Background

The term burnout was first used in the clinical setting in 1974 by the psychologist Herbert Freudenberger, who carried out a study on the change of attitude of health personnel relating burnout with states of anxiety and depression, considering it “a set of non-specific medical-biological and psychosocial symptoms that develop in work activity as a result of an excessive demand for energy” [36]. From this point, the research stage in Burnout begins [37], characterized by the first clinical analyses of the syndrome and the ratification of its regularity. Later on, Cristina Maslach elaborates a representation of the responses of the workers related to the care environment, when they feel vulnerable due to emotional stress [38]. In 1981, the social psychologist Christina Maslach defined it as “a three-dimensional syndrome characterized by emotional exhaustion, depersonalization and low personal realization that can occur among individuals who work in direct contact with clients and/or patients” [39]. Together with Dr. Jackson, they created one of the most widely used instruments to measure burnout: the Maslach burnout inventory (MBI) by evaluating the three dimensions mentioned.

The most vulnerable professionals to suffer from burnout are those who develop their work in relation to other people such as health and teaching professionals [27,40,41].

Studies on burnout in health professionals have been conducted on numerous occasions relating symptoms of mood disorders, anxiety, and depression as this syndrome consequences [42,43,44]. Its effects are usually evident within a depressive symptomatology which scope transcends the merely occupational scenario and involves the personal, family, and social sphere [45,46].

Under normal circumstances, the health profession generates work-related stress or burnout for a variety of reasons, including continuous contact with pain and suffering [47,48], as well as monotonous tasks, heavy or excessive workloads, the frequency and amount of time spent with patients, and even work development and management styles [49,50]. On the other hand, there are more personal factors such as one’s ability to cope with death and suffering and poor or bad interpersonal relationships, which can also be a cause of stress and psychological distress.

In this sense, and taking into account the clinical point of view, it is considered that work stress, characterized by workload, time pressure, demands, resources or management control, among others, can trigger burnout syndrome (as a state). However, the psychosocial approach considers that it is the work and personal environment that can trigger the appearance of this syndrome (as a process) [51], i.e., it arises as a response to labor stress [52] without there being an exact cause that precipitates it, but the realization of which is not capable of minimizing the dissatisfaction or lack of motivation that is present.

Maslach defined burnout as “a chronic stress produced by contact with clients that leads to emotional exhaustion and alienation from clients in their work” [53]. This definition was widespread during the decades of the late twentieth century [54,55]. Today, however, burnout is defined as a “prolonged response to chronic personal and relational stressors at work, determined from dimensions known as burnout, depersonalization, and professional cynicism and inefficiency” [45].

In the face of a threat such as that posed by COVID-19 disease, as well as other special emergency situations, where rapid action is required and the workload is high, it often happens that the primary reaction is to act, without thinking about the individual’s emotional needs. In contrast, we can find protective factors that improve professional practice. In this respect, a recent study by Kisely et al. showed that “clear communication, access to adequate personal protection, adequate rest and both practical and psychological support were associated with a reduction in morbidity” [56].

At the first stage of exposure to the virus, health professionals had to make a great effort, which usually leads to anxiety and consequently the possible intake of anxiolytics, to maintain a constant capacity for work, which could lead to depression on an ongoing basis and after a few months of maintaining this habit, a post-traumatic stress disorder as occurred in other epidemics such as SARS, MERS, influenza A/H1N1 or Ebola [57,58].

At a physical level, burnout syndrome is associated with the appearance of certain disorders that often force the affected person to request sick leave from work [59,60], such as high muscle tension and generalized musculoskeletal pain (fibromyalgia), headaches or backaches, central nervous system dysfunctions, sexual dysfunctions or various cardiovascular and gastrointestinal problems [61]. 

All these elements guide the need to study the effects that the current pandemic may be having on health professionals from the different subscales of burnout, and thus enable a learning process to prevent possible future situations similar to the COVID-19 pandemic or new waves of this same virus. The World Health Organization [62,63] and many researchers notice the importance of initial and continuing training for health professionals [64,65,66,67,68], especially in the context of emergency situations [69,70,71,72].

In this context, training for mental health professionals in these extreme scenarios, which have a huge psychological impact, is essential [73,74,75]. Most studies focusing on this problem highlight that training and prevention processes can reduce the incidence of burnout syndrome in health professionals [76,77,78,79,80,81,82,83,84]. It is even argued that a combination of actions (including organizational, support and changes in the work environment) can have an even greater effect [85,86].

However, most of these contributions have focused on the prevention of burnout syndrome in everyday work situations. Although there are studies on professionals working in emergency services [87,88,89,90,91,92], which would offer similar results, we lack more knowledge when it comes to borderline situations in disasters and catastrophes contexts. These include a global pandemic, a global health emergency that has been unknown for almost a century, when the terrible Spanish flu of 1918–1919 occurred [93]. It is, therefore, necessary, prior to develop programs designed for this purpose, to know the impact on health workers, of a situation of such stress and tension, caused by the COVID-19 epidemic in its most extreme phases.

In this sense, different studies have found that along with service sector workers and teachers, physicians and nurses had high burnout rates [27,94], related to job dissatisfaction, little control over work, lack of recognition, low pay and caring for people in a terminal situation [62,95]. It is, therefore, necessary to establish a description of the state of health professionals, in relation to this important issue, at the most critical moments of the first wave of the pandemic. We are at a critical moment and this data can be very valuable, especially when thinking about the future of health professionals and comparative studies can determine an evolution in this area.

## 3. Materials and Methods

### 3.1. Objectives

In the health emergency caused by COVID-19 disease, the occupational stressors and anxiety of health professionals could have been aggravated by the virulence of the pandemic and the shortage of PPE. In this context, the aim of this research is to find out how the current health crisis has affected health professionals, considering the Maslach and Jackson burnout subscales of the Maslach burnout inventory [55], adapted by Seisdedos [96] and validated by García, Herrero y León [97], during the most critical weeks of the spread of the virus. We decided, therefore, to undertake a study that would describe the situation at that specific moment, in the absence of research at the time, although after this research was carried out, works with similar objectives have been published [98,99] which reinforce the need for knowledge of this problem. In this way, the object of study was centered on the appearance of burnout in health centers in situations of need for an immediate and effective approach, as is the case of the COVID-19 pandemic, where the lives of many patients were in serious danger. This information would be very valuable for the construction of future prevention protocols and training of healthcare personnel in the face of pandemics of these characteristics or borderline scenarios.

### 3.2. Study Design and Sample

The research has been carried out in collaboration with health personnel working in hospital centers in Spain, mainly nurses, physicians, and nursing assistants. The subjects studied were 157. The period in which the questionnaire was administered was between April 6 and 19, in the middle of the confinement stage and where daily deaths and active cases of contagion reached their peak in Spain. Although the sample can be considered small compared to other studies, the need to carry out this research at this historical moment made the benefits greater than the disadvantages. For this reason, it was not possible to carry out research based on a representative sample adjustment in the whole of Spain, but rather an intentional cut-off methodology was followed. 

To gain access to health professionals who worked directly with patients affected by COVID-19, representatives of workers, hospital and ICU staff from different Spanish hospitals were contacted directly, who facilitated contact with the workers under study. For this reason, the sample is smaller than what could have been obtained by extending the research to all health professionals. In any case, this methodology made it possible to previously select the subjects who were really working with the disease during this period. Subsequently, an online survey was administered through the application of the University of Murcia (umu.encuestas) to which the participants had access without putting their security at risk, and always guaranteeing their confidentiality and anonymity. Participants must accept the ethical conditions and give their consent before accessing the questionnaire and sending their answers.

In any case, all the requirements of the Ethics Committees that Spanish universities establish for their researchers were followed. Since this is a descriptive study in Spain, the approval of a specific Ethics Committee was not required (unlike studies based on experiments). Despite this, the Codes of Good Practice for Research on Human Beings were followed. Furthermore, it is important to add that the participants gave their informed consent in accordance with the Declaration of Helsinki. The project was registered and signed by the research team with the code number REPRIN-PEM-010. 

The research design was a simple random sampling in view of the difficulties aroused from accessing health professionals in such a short period of time-due to the phase of confinement in which the Spanish population found itself after the state of alarm decree—and the need to obtain relevant data on this social phenomenon in such an important historical context.

### 3.3. Variables Used

#### 3.3.1. Dependent Variable

The Maslach burnout inventory (MBI) was used to measure the burnout. This questionnaire was validated by Maslach and Jackson and the final version was published in 1986. A validated and translated version of the original was used, which has already been used in numerous studies in Spain with positive results [60,100,101,102,103,104,105,106,107]. This is a 22-item questionnaire with 7 answer options (Likert scale from 0 to 6) which comprehends the following subscales: emotional exhaustion (EE), understood as the subject’s feeling of being emotionally saturated by work; depersonalization (DP), which implies a cold and impersonal response to patients; and personal accomplishment (PA), which encompasses feelings of competence and efficiency at work. With respect to emotional exhaustion, the scores of the different levels are as follows: 0–18 for the low level, 19–26 for the medium level, and 27–54 for the high level. In relation to depersonalization, the low-level ranges from 0–5, the medium level from 6–9, and the high level from 10–30. Finally, regarding personal accomplishment, the scale attributes a low level when values between 0–33 are registered, a medium level when they are between 34–39, and a high level when values between 40–56 are obtained.

In each of the three dimensions, the dependent variable has been established as dichotomous, differentiating between low and medium/high risk. For this purpose, the reference values in each of the subscales were followed to categorize the dependent variable. The medium–high risk in each of the subscales is as follows: emotional exhaustion: 16–54, depersonalization: 6–30 and professional accomplishment: 34–56.

#### 3.3.2. Independent Variables

The following variables were used to predict the burnout: (a) sex (male/female), (b) age (<30 years, 31–40 years, 41–50 years, 51–60 years and >60 years), (c) professional category (nurse, doctor, clinical assistant and others), (d) perception of need for psychological/psychiatric treatment (yes/no), (e) perception about the need to incorporate psychological/psychiatric treatment (yes/no), (f) perception about whether they think they will need psychological/psychiatric treatment in the future (yes/no), (g) if they feel that the lack of PPE may be affecting their level of stress or anxiety (yes/no).

### 3.4. Statistical Analysis

Firstly, a descriptive analysis of the most representative variables was developed considering sociodemographic aspects and perceptions of stress and anxiety of health professionals. Subsequently, a cross-table analysis was developed with the aim of observing the relationships between the different variables according to their level of significance (*p* < 0.005).

Finally, with the purpose of knowing the probability of the phenomenon of the three subscales of burnout, three logistic regressions were carried out, establishing as a dependent variable in each one of them the risk of suffering a medium-high level of burnout, taking into account the independent variables.

## 4. Results

The descriptive analysis shows the following results (Table 1). First, with respect to sociodemographic variables, 79% are women and 21% men; the percentage of female participation is higher. This has occurred with other studies with very similar percentages [108,109,110]. The mean age is 41.8 years. If we make a distribution by age, the sample is widely distributed among all the intervals except among people aged 61 years and older. In terms of professional category, the nurses who accounted for more than 50% of the participants are noteworthy.

As for the MBI scale, the average value is 3.3. If we carry out a more detailed analysis of the MBI scale, we observe that the highest values are concentrated in the depersonalization subscale whose high levels reach 38.9%, almost doubling the values of the EE and PA subscales. Finally, with respect to the data related to subjective issues, the following data were obtained. In the first place, only a small percentage of health professionals consider that they need psychological and psychiatric support, but, nevertheless, 90.4% of the persons surveyed consider that this service should be provided from the work centers in view of the current health crisis.

Related to the above, 43.3% of the persons surveyed considered that they might need psychological or psychiatric treatment in the future. In other words, health professionals do not currently perceive a situation of emotional vulnerability, but they are aware of the intensity of it and the repercussions it may have for them in the future. Finally, when asked how the absence of PPE is affecting their daily work, 85.4% stated that it is generating an increase in their level of stress and anxiety.

Once the descriptive analysis was done, a cross-table analysis was performed. Before performing this analysis, a classification of burnout risk variables was carried out in each of the subscales of the MBI, differentiating between low/medium risk and medium/high risk.

The results that obtained a Pearson chi-square with a significance level *p* < 0.05 will be presented; that is, those that show a significant relevance and whose association is not determined by chance.

First, it highlights the association between emotional exhaustion and the subjective perception of needing psychological or psychiatric treatment in the future. Regarding this subscale, a significant relationship is also observed between the risk of suffering emotional exhaustion and the professional category. Second, with respect to depersonalization, we observe how the lack of resources of individual protection is associated with this subscale. This result is very interesting given that during the first wave of the pandemic, health professionals had to carry out their work with insufficient resources of individual protection, putting their personal and family health at risk. This fact has important connotations since the lack of individual protection equipment could have caused an increase in the subscale of depersonalization, and with it, cause a detachment of health professionals from their problems, even to blame their patients for the problems caused by the COVID-19 at the professional level. With respect to personal accomplishment, no significant associations were observed. Therefore, the three independent variables that most significantly influence the increase in the level of burnout, taking into account the level of significance of Pearson’s chi-square, are: professional category, subjective perception of the need to receive psychological or psychiatric treatment in the future, and the absence of PPE.

Thirdly, we proceeded to use the technique of binary logistic regression to evaluate the effect of sex, age, professional category and subjective perceptions regarding the present and future needs for psychological and psychiatric treatment in health professionals; if this should be provided by hospital centers, as well as if the lack of resources of individual protection is affecting the increase in burnout in health professionals (Table 2). The only subscale that showed an acceptable model was emotional exhaustion.

The logistic regression model was statistically significant, X2 = 29.942, *p* < 0.0005. The model explains 24.6% (Nagelkerke’s R2) of the variance in the emotional exhaustion subscale and correctly classifies 75.2% of the cases. The Hosmer–Lemeshow test showed that there were no significant differences between the results observed and those predicted in the model with a *p* = 0.830.

From these results, the less significant variables were eliminated through the automatic “forward” or “backward” method. Considering the seven predictor variables, only two were associated with the probability of suffering emotional exhaustion: the professional category and the subjective perception of needing psychological or psychiatric treatment in the future (WNMS). Regarding the professional categories, physicians and nurses have a higher risk of suffering from emotional exhaustion than other professions, including clinical assistants, professionals who in Spain spend the most time with patients performing the most elementary care with basic needs. The results of the logistic regression are shown below (Table 3).

In the specific case of physicians, the OR was = 4.270, 95%CI (1.180–15.445), *p* = 0.027. Nurses had an OR = 3.782, 95%CI (1.419–10.078), *p* = 0.008. Therefore, physicians and nurses are at risk of suffering from emotional exhaustion. Their risks are 4.27 and 3.78 times greater than other health professionals, respectively. In case of both, physicians and nurses, the contact with patients and the time they spent with them was superior to that of other health professionals, and it was these same professionals, during the time of the greatest number of active cases of COVID-19, who were responsible for trying to restore the health of the patients, at the risk of infecting themselves due to the shortage, in the first few weeks, of personal protective equipment. Likewise, the fact of seeing their patients die every day has been key to the realization that, in the future, when the situation improves, they would need to address this issue in the personal sphere, with professional intervention at the psychological and/or psychiatric level.

Finally, in relation to the subjective perception of needing psychological and psychiatric treatment in the future, the data show OR = 0.244, 95% CI (0.109–0.547), *p* = 0.001. Therefore, and contrary to what might initially be thought, the probability of a person who considers that they may need psychological and psychiatric treatment in the future decreases by 0.24 with respect to those who do not perceive that they consider these treatments necessary. This result suggests that, despite a future perception of the need for psychological treatment, the health demands caused by the COVID-19 pandemic focus their attention on patient care, over and above their own concerns. High-stress situations, in which health personnel do not perceive the need for psychological care, reinforce the fact that they behave homogeneously in the context of this pandemic and that sociodemographic conditions, such as age and sex, are not highlighted. This is essential information that we believe must be considered in any future preventive planning to face these harsh adversities. Prevention is a key factor.

## 5. Discussion

The results demonstrated us how the burnout syndrome is currently affecting more those aspects related to depersonalization than the rest of the subscales of the MBI. Unlike other studies [15,111,112,113,114] it is striking that, during this health crisis, no differences are seen in relation to sex and age as it does on non-exceptional occasions, where it most affects older female health professionals [115].

On the other hand, in relation to the depersonalization scale, we observed a relationship between people who present higher levels in this subscale as a result of the absence of PPE and their subjective perception that it can produce an increase in stress and anxiety levels. Recent studies related to mental health in the face of the COVID-19 pandemic address this phenomenon, demonstrating the fact that health workers face challenges such as the risk of infection, excessive care burden, exposure to family distress and ethical and moral dilemmas [23,57].

This is not a trivial issue, nor is the fact that during the most critical weeks for the health care system, where daily deaths reached almost 1000 people and new infections were counted in the thousands, health care workers lacked PPE, posing a risk to their own health as a group, a point that concurs with the study by Xiang et al. These elements are key to understand that increased anxiety as a result of gaps in pandemic planning and prevention has had an impact on health workers in their personal, family and community spheres, recording burnout values above those found in Spain in previous times [27,115,116]

Moreover, in a remarkable way, in the subscale of emotional exhaustion, we observed from the logistic regression carried out, the higher probability of suffering burnout concentrates in physicians and nurses over other health professionals. The fact that they surpass the clinical assistants, personnel who carry out the most elemental care of the patients, stands out. However, this risk of suffering emotional exhaustion is not the result of the lack of cohesion between the different professional categories, but rather of the lack of foresight from competent professionals regarding the general lack of individual protective equipment among health professionals, especially those who were most exposed to the virus due to increased contact with patients. This can be understood within a context where emotional exhaustion is linked to the capacity to respond to the disease from a more scientific perspective than that of companion and care. In this sense, the work done would be aimed, mainly in certain units such as the intensive care unit, where the demand for beds was higher than the existing capacity, to save the lives of infected patients who, although many could be saved, many others did not. That is to say, the absence of an effective response to COVID-19 disease on the part of these professionals has led to greater emotional exhaustion. Similar results were found in recent studies such as that of Li et al. [117] which indicate that physicians in China experienced an increase in mental health symptoms and fear of violence and a decrease in mood after the outbreak of COVID-19.

On the other hand, a singular fact is taking place in relation to the subjective perception of the need to receive psychological and psychiatric treatment. In this case, the people who have a greater risk of suffering emotional exhaustion are precisely those who, at the time of carrying out the study, consider that they will not need it, a sign of the pressure in which they currently work. This finding is analogous to the results found by Chen et al. [118] who expressed that health professionals were concerned about the insufficiency of PPE and feelings of helplessness when faced with critical patients. Many indicated that they did not need psychological help, but rather more uninterrupted rest and sufficient protective equipment. 

It is not difficult to think that this situation will not have repercussions on professionals in the long term, since on many occasions the individual himself is not prepared to face limited situations at the time of greatest tension; often these consequences appear in the future. As Albaladejo and others point out [111], after 10 years from the beginning of a stressful situation, a process of sensitization or de-motivation can occur, losing the vocation that, at the time, made the person choose this profession.

At this point, professional treatment can have significant benefits for the individuals concerned, producing both personal and occupational rewards. In this particular case, characterized by an unprecedented health crisis where there has been a lack of adequate response from health institutions in providing essential PPE to their workers, psychological treatment emerges as a plausible and reasoned need for those health workers who believe they may need this treatment in the future.

In other words, health professionals who do not perceive the need for these treatments, in the face of the adverse health crisis situation, are what may need them most in the future since their emotional exhaustion is increasing without obtaining any compensatory mechanism for the situation of stress and emotional anxiety. Studies such as that previously cited by Chen et al. [106] found similar data; at the first stage of the illness, health professionals were reluctant to participate in the group or individual psychological interventions provided to them for various reasons: becoming infected was not the main concern when they began to work; they were more concerned about the possibility of their relatives worrying about them and about the possibility of bringing the virus home; and finally, the health workers did not know how to act with patients who did not want to be quarantined in the hospital, sometimes because of the panic that ignorance of the disease caused and they did not collaborate with the medical measures. 

In this sense, in an emergency context such as the one that occurred during the key months of the COVID-19 outbreak, the measures carried out and the prevention actions at the beginning of the process are more effective. In relation to this issue, Withey [119] considers an emergency situation as a type of stress in which the appearance of several factors lead to the existence of different degrees of fear, anxiety and concern that result in adaptive or non-adaptive efforts; these factors are: the severity of the event, the probability of occurrence and the individual’s ability to cope with the crisis and the existing stress. Factors existing during the coronavirus outbreak.

It is also worth asking whether there could be training, both initial and ongoing, for health professionals in dealing with these extreme contingencies, in such a way as to enable them to face these situations with techniques to control their stress, fear and emotions. Academic offerings of specific subjects during the university career or training modules in continuous training processes could be a possibility, but the experiences obtained so far in other scenarios of extreme circumstances do not show that satisfactory results can be guaranteed. As presented by Dreison et al. [76], in a meta-analysis of the effectiveness of interventions against burnout syndrome, the effectiveness is very relative. It is true that training/education appeared in their study as the most effective subtype of organizational intervention, but this was dependent on the context. These authors even recommended that the different interventions carried out be adjusted to the specific needs of the organization and staff, and in long follow-up periods.

However, as we have presented in this study, the situation in which Spanish health professionals have found themselves in this COVID-19 pandemic is what has led to the burnout syndrome having more effect on those aspects related to emotional exhaustion. Further, this has been due, above all, to the fact that they lacked the resources to deal with the virulence of SARS-CoV-2 rather than the lack of prior training or preventive measures in training. The lack of resources of individual protection was one of the highest risks that health professionals have had to face in their professional work, being aware that they were risking their lives and, naturally, those of their loved ones, to whom they could infect. Nevertheless, the work carried out has been admirable, always focusing on the care of patients above their own concerns. In this context, the appearance of the burnout syndrome is evident. If the situation caused by the pandemic has significantly increased the levels of anxiety and stress in the general population, for which interventions are requested [120,121,122], the experiences of health professionals in Spain, faced in the front line with the most terrible aspects of this disease, and in so many cases without sufficient protection, represent a limited scenario on the psychological level. 

Thus, in relation to its meaning, is necessary to emphasize that, unlike the subscales of emotional exhaustion and depersonalization, no significant results were observed in terms of personal accomplishment, neither through the cross tables nor through logistic regression. This is a relevant finding since it can be considered that the previous variables that health professionals highlight as generating stress and anxiety are the lack of PPE and the possible transfer of the virus to relatives and not so much the excessive workload or lack of motivation, which, according to the data, appears intact, despite being in an unprecedented health crisis.

Finally, it should be noted that this research was limited by the accessibility to health professionals at that critical time as a result of the declaration of the state of alarm and the extreme conditions in which they carried out their job. Despite not having developed a stratified research in all the Spain territory, and with a greater sample (the mentioned limitations made it impossible) the results allow access to information of vital importance both for the current management of the health crisis and to design, plan and execute actions to improve the mental health of the health professionals subjected to high levels of anxiety and stress at work. In the context, as we say, of how difficult it has been to carry out the field work, we consider it necessary and essential for all that it could bring us.

Understanding depersonalization as the unemotional and impersonal response to patients, development of negative attitudes and feelings, insensitivity, distancing of patients and colleagues even blaming them for their own frustrations, we find that in this study, the main finding lies in the relationship with the lack of resources of individual protection and the risk of health care workers to be infected and not as a response to fatigue resulting from high workload, time with patients or lack of recognition. In this case, changes in management related to schedules, work shifts, service rotation, training in effective stress management and conflict resolution, tolerance to frustration, as well as the importance of companion and quality of care, would help to minimize this feeling of tiredness and cynicism.

## 6. Conclusions

This article shows the need to consider mental health care for the health care community, especially those who have been in the front line, working with confirmed and suspected cases of COVID-19, to avoid possible malpractice, but also the development of post-traumatic stress in both health and non-health care extending also to patients who may need it, especially by multidisciplinary teams and in those at risk of suicide. 

Up-to-date, regular, and accurate information is essential both, to reduce the high levels of burnout in physicians and nurses and to avoid the feeling of fear and uncertainty that occurred especially in the first weeks of official coexistence with the virus. Both physicians, and nurses have been the health occupations most affected by burnout, precisely those who are most connected in the detection and treatment of the disease. Attention to symptoms such as insomnia, social isolation, information on self-recognition of stress symptoms [123] would have been essential from an institutional intervention. The guidelines adopted and proposed at international level can serve as a guide for possible outbreaks of coronavirus in the healthcare context. It is essential to anticipate what may happen [56,124,125,126].

Identifying the lack of means of individual protection and the danger of health workers to be infected, with the depersonalization that health workers present, is one of the main contributions of this study since it justifies and relates these reactions of health workers to the work carried out during the COVID-19 pandemic. Therefore, these aspects, which were not avoided and generated a high stress due to the real possibilities of contagion and death, are closely related to the unwanted attitudes inherent to the state of depersonalization of professionals.

This work has some limitations such as reaching a larger sample. However, it must be taken into account that the applied research was carried out in a context of a high level of difficulty in administering the questionnaire to health professionals—submerged in a situation of work stress not known until now—as well as the need to obtain results at the moment of greatest virulence of the COVID-19 disease, it being appropriate not to miss the opportunity to carry out this study at this historical moment. Likewise, and related to the previous limitation, we find the difficulty of carrying out a sample stratification by territories that would have allowed us to analyze the results according to the health policy implemented in each one of them. It would have been convenient to compare the results with other different questionnaires used to measure the existence and level of burnout in health professionals (for example, GAD-7 or PHQ-9), but due to the time pressure and the impossibility of having access to more health professionals due to the conditions they are experiencing, it was not possible. However, there has been a several number of publications on burnout in health professionals in Spain that used the MBI scale which gives consistency and supports the results of this study [127,128,129].

## Figures and Tables

**Table 1:** Descriptive analysis of the variables.

	*N*	%	Average
**Gender**			
Woman	124	79.0	
Man	33	21.0	
**Age**			41.8
<30	35	22.3	
31–40	40	25.5	
41–50	30	19.1	
51–60	36	22.9	
>60	16	10.2	
**Job**			
Doctor	22	14.0	
Nurse Assistant	29	18.5	
Nurse	80	51.0	
Other	26	16.5	
***IMBI Scale Total***			
**Sub Emotional Exhaustion**			
Low	92	58.6	
Medium	33	21.0	
High	32	20.4	
**Sub Depersonalization**			
Low	50	31.8	
Medium	46	29.3	
High	61	38.9	
**Sub Personal Accomplishment**			
Low	72	45.9	
Medium	54	34.4	
High	31	19.7	
**Need Support P/P**			
Yes	42	26.8	
No	112	73.2	
**Support Should be Given**			
Yes	142	90.4	
No	14	9.6	
**He/She’ll Need Support P/P**			
Yes	68	43.3	
No	87	56.7	
**Absence of PPE, Increases Stress/Anxiety**			
Yes	134	85.4	
No	20	14.6	

**Table 2:** Variables used in binary logistic regression.

**1. Gender**
Ref. Man
(1) Woman
**2. Age**
Ref. < 30
(1) 31–40
(2) 41–50
(3) 51–60
(4) >60
**3. Job**
Ref. Other
(1) Nurse
(2) Doctor
(3) Nurse Assistant
**4. Needs Psychological/Psychiatric Support**
Ref. No
(1) Yes
**5. Psychological/Psychiatric Support Should be Provided at Your Workplace**
Ref. No
(1) Yes
**6. He/she will Need Psychological/Psychiatric Support**
Ref. No
(-1) Yes
**7. Increased Stress and Anxiety as a Result of Lack of PPE**
Ref. No
(1) Yes

**Table 3:** Results of logistic regression.

	B	Sig.	Exp(B)	95% C.I. for Exp(B)	
				Lower	Superior
WNMS (1)	−1.412	0.001	0.244	0.109	0.547
Job		0.049			
Nurses	1.330	0.008	3.782	1.419	10.078
Physicians	1.452	0.027	4.270	1.180	15.445
Nurse Assistant	0.926	0.119	2.524	0.787	8.089
Constant	0.650	0.175	1.916		
